# Textbook outcome can be reached in the learning curve for hybrid robot-assisted esophagectomy–experience from a German high-volume center

**DOI:** 10.1007/s11701-026-03405-6

**Published:** 2026-04-21

**Authors:** Nicolas Jorek, Ali Majlesara, Leonard Klevesath, Nerma Crnovrsanin, Ingmar Rompen, Frank Pianka, Arianeb Mehrabi, Christoph W. Michalski, Henrik Nienhüser

**Affiliations:** Medical Faculty Heidelberg, Department of General, Visceral and Transplantation Surgery, Heidelberg University, Heidelberg University Hospital, Im Neuenheimer Feld 420, 69120 Heidelberg, Germany

**Keywords:** Esophagectomy, Esophageal cancer, Robot-assisted minimally invasive esophagectomy (RAMIE), textbook outcome, learning curve

## Abstract

Esophageal resections are associated with significant postoperative morbidity and mortality. The standard surgical approach is minimally invasive, with a high proportion of robotic resections. The aim of this study was to evaluate the learning curve (LC) in the context of establishing a structured training program. As part of the retrospective analysis, all consecutive hybrid robot-assisted Ivor-Lewis esophageal resections performed by a defined team between May 2023 and October 2025 were evaluated. The LCs for the frequency of postoperative complications Clavien-Dindo (CD) ≥ III, in particular anastomotic leakage, and the textbook outcome rate were analyzed. The LCs for these target variables were evaluated using the cumulative sum (CUSUM) analysis. Between May 2023 and October 2025, a total of 71 patients underwent hybrid robot-assisted Ivor-Lewis esophagectomy. In-hospital mortality in the study population was 1.4% (1/71). Postoperatively, 29.6% (21/71) of patients experienced postoperative complications classified as grade IIIa or higher according to CD. A total of 9.9% of patients (n = 7) developed anastomotic leakage. After case no. 20 a significant reduction in anastomotic leakage rate was observed, with a leakage rate of 2% after completion of the LC. The rate of textbook outcomes in our cohort was 49% (35/71). The LC for robot-assisted esophageal resections is associated with the occurrence of postoperative complications. After 44 operations there is a reduction in complications. Total in-hospital mortality was low, demonstrating that robotic procedures can be learned safely at experienced centers while achieving textbook outcomes during the learning curve.

**Supplementary Information:**

The online version contains supplementary material available at 10.1007/s11701-026-03405-6.

## Introduction

Esophageal cancer currently ranks as the seventh leading cause of cancer-related mortality worldwide [1]. Oncological resection in a multimodal setting is considered the standard of care for locally advanced, non-metastatic esophageal cancer [2]. Typically, a transthoracic resection with a 2-field lymph node dissection according to Ivor-Lewis is performed [3]. Yet, overall 5-year survival remains poor, with reported rates below 25% [4]. Furthermore, esophageal resection is associated with significant postoperative morbidity and mortality [5, 6].

Robot-assisted minimally-invasive esophagectomy (RAMIE) was introduced in 2003 and has seen a steady increase in clinical application since then [7, 8]. In comparison with open resection, RAMIE has been demonstrated to reduce both overall and cardiopulmonary postoperative complications without compromising oncological outcomes [5, 9, 10].

However, for the implementation of a robotic program in a demanding procedure like esophageal resection an initial learning curve (LC) has to be overcome. This initial period is associated with significant morbidity- especially anastomotic leakage [11]. In the surgical field, the LC is a term used to denote the requisite number of cases to attain proficiency with regards to a certain outcome variable.

In recent years, several analyses of the LC in the adoption of RAMIE have been published. The hypothesized case load for overcoming the LC exhibits significant variability, ranging from 9 to 85 cases [12]. However, many of these publications employed only arbitrary statistical methods and did not report on prior surgical experience [13]. Furthermore, operating time, blood loss and R0 resection rates were the primary metrics utilized for LC evaluation, with only a limited number of studies targeting complications or anastomotic leakage rates as their outcome variables [14, 15].

The aim of our study was the evaluation of the LC of a defined surgical team during their training for esophageal cancer surgery at our clinic. Therefore, the LC for the first 71 consecutive Ivor-Lewis hybrid RAMIE (hRAMIE) cases was analyzed for postoperative complications defined as Clavien-Dindo (CD) ≥IIIa, anastomotic leakage and textbook outcome rate. Furthermore, the LC for duration of surgery, intraoperative blood loss, lymph node yield, intensive care unit (ICU) stay, and length of hospital stay was evaluated.

## Methods

### Study design

This study is a retrospective, single-center cohort analysis of the LC during the training of a surgical team for robotic Ivor-Lewis resections with intrathoracic anastomosis. Ethical approval was obtained from the Ethics Committee of the Medical Faculty of Heidelberg (S-649/2012). This study was conducted in accordance with the STROBE (Strengthening the Reporting of Observational Studies in Epidemiology) guidelines [16].

### Patient cohort and data retrieval

Between May 2023 and October 2025, 71 consecutive patients underwent hRAMIE with intrathoracic anastomosis at the Surgical Department of the Heidelberg University Hospital. All operations were performed by a single surgical team with HN as the console surgeon and CM and AM providing guidance and oversight. CM and AM had extensive previous experience in robotic as well as open major abdominal surgery. For training of the console surgeon, a structured training program using a stepwise approach was conducted under supervision. Esophageal resection was divided into 20 single steps of different complexity varying from easy to moderate to challenging (See Supplement 1). These steps were then performed under supervision using a step-up approach according to the assigned degree of difficulty.

Clinical data was retrieved from a prospectively managed database at our surgical clinic. This data set included demographic characteristics, comorbidities, oncological data, and postoperative outcome parameters. A comprehensive review of the anesthesiology files was conducted to retrieve intraoperative data, including the duration of surgery, intraoperative blood loss and number of blood transfusions. Preoperative confirmation of all tumors was conducted through histopathological analysis. The indication for surgical resection was confirmed by our interdisciplinary tumor board according to the German oncologic guidelines. Patients underwent hRAMIE using the DaVinci Xi system (Intuitive Surgical, Sunnyvale, CA). Our detailed technique for the robot-assisted abdominal phase has been previously described [17]. For the thoracic phase a right anterolateral thoracotomy was performed. Anastomosis was performed in end-to-side configuration using a circular stapler in all cases, favoring a larger 29 mm stapler where possible. A nasogastric tube, a chest tube (24Ch) and an abdominal Robinson drain (16Ch) were placed intraoperatively. Exclusion criteria for hRAMIE were previous extensive abdominal surgery, due to an increased risk of adhesions. Criteria for transfer to the regular ward were as follows: more than twelve hours of stable circulation without vasopressors, a heart rate of 50–110/min, maximum nasal oxygen flow of 4 L/min, no vomiting as well as delivery rate of the nasogastric tube < 400 ml/d and successful mobilization (walking without support).

Postoperative complications were classified according to the CD classification system [18]. Complications classified as CD ≥ IIIa were regarded as major complications. Anastomotic leakage was defined as a full-thickness defect of the anastomosis according to the Esophagectomy Complication Consensus Group (ECCG) [19]. Diagnosis for AL was confirmed endoscopically and by computed tomography. For measurement of textbook outcome the definition by Busweiler et al. was utilized, warranting surgical and pathological R0 resection, no intraoperative complications, retrieval of at least 15 lymph nodes, no major postoperative complications, no reintervention, no readmission to the ICU or IMC, no hospital readmission after discharge, no postoperative mortality and a hospital stay of 21 days or less [20]. Notably, for comparison with the UGIRA data the textbook outcome definition was altered utilizing CD ≥ III instead of CD ≥ II as an exclusion criterion [21]. Postoperative patient follow-up after discharge was conducted up until postoperative day (POD) 90.

### Statistical analysis

Continuous variables are presented using either the mean with standard deviations or median with interquartile ranges (IQR). Categorical variables are displayed using frequencies and percentages. To compare differences of clinical parameters groups, two-sided students t-test was used for normally distributed continuous variables and Fisher’s exact text was used for categorical variables. The Mann-Whitney-U test was used for non-normally distributed variables. P values < 0.05 were considered statistically significant. Statistical analysis was performed using RStudio with packages.

In order to assess the LC for robot-assisted esophagectomy we performed CUSUM analysis for different surgical outcome variables. The “cusum” function from the “qcc” package in R was utilized for this analysis. The reference values employed for the calculation of the respective CUSUM plot included the benchmark complication rate of 31% reported by Low et al. for CD IIIa or higher, the textbook outcome rate of 51% after completion of the LC from the Upper GI International Robotic Association (UGIRA) Esophageal Registry, and the anastomotic leakage rate of 18.6% reported by Weber et al. from the German Diagnosis Related Groups (G-DRG) statistics [8, 21, 22]. For each CUSUM plot, individual control limits were calculated to evaluate the time point at which the initial LC was completed. For continuous variables control limits could not be calculated. Therefore- as previously described by several authors- we graphically estimated the inflection point of the CUSUM plot- indicating overcoming of the LC [14, 15]. As the reference value we used the intrinsic mean of the respective variable.

## Results

### Patient characteristics

Between May 2023 and October 2025, a total of 71 patients underwent hRAMIE Ivor-Lewis at our institution. The median age of our study population was 65 years (IQR: 60–71 years), with a majority of male patients (85%). Median BMI was 25.3 kg/m² (IQR: 23.1–28.9 kg/m²). A significant proportion of the patient population exhibited an ASA Score of three (63%), with the majority of these cases attributable to either cardiovascular (63%) or pulmonary risk factors (24%). The presence of malignant neoplasms was the indication for surgical treatment in all cases. A total of 58 patients were preoperatively diagnosed with adenocarcinoma of the esophagogastric junction, 10 with squamous cell carcinoma, and three with neuroendocrine carcinoma of the esophagus. 62 out of 71 patients (87%) received neoadjuvant treatment mainly according to the FLOT or the CROSS protocol (61% and 17% of patients respectively) (see Table 1).

**Table 1:** Baseline patient characteristics of the study population

Patient characteristics	*n* = 71
Age [years, median (IQR)]	65 (60–71)
Male [n (%)]	60 (85)
BMI [kg/m², median (IQR)]	25.3 (23.1–28.9)
ASA stage [n (%)]	
I + II	26 (37)
III	45 (63)
Charlson comorbidity index [median (IQR)]	3 (2–4)
Cardiovascular risk factors [n (%)]	45 (63)
Pulmonary risk factors [n (%)]	17 (24)
Diabetes [n (%)]	10 (14)
History of smoking [n (%)]	35 (49)
History of drinking [n (%)]	10 (14)
Diagnosis	
AEG [n (%)]	58 (82)
SCC [n (%)]	10 (14)
Neuroendocrine carcinoma [n (%)]	3 (4)
Neoadjuvant treatment	
FLOT protocol [n (%)]	43 (61)
CROSS protocol [n (%)]	12 (17)
Others [n (%)]	7 (10)
None [n (%)]	9 (13)

The median duration of surgery was 282 min (IQR: 248–346 min) with median blood loss of 350 ml (IQR: 250–500 ml). Four patients (5.6%) received intraoperative blood transfusions. Conversion to laparotomy occurred in one case due to the presence of intraabdominal adhesions (see Table 2).

**Table 2:** Intraoperative outcome parameters

Intraoperative outcomes	*n* = 71
Duration of surgery [min; median (IQR)]	282 (248–346)
Blood loss [ml; median (IQR)]	350 (250–500)
Blood transfusion [n (%)]	4 (5.6)
Conversion rate [n (%)]	1 (1.4)

The median length of hospital stay for patients was 19 days (IQR: 15–26 days), with a median ICU stay of one day (IQR: 1–2 days). The in-house-mortality rate was 1.4% (1/71) and remained consistent at POD 90. Postoperatively, 29.6% (21/71) of patients experienced postoperative complications classified as CD grade IIIa or higher. The prevalence of anastomotic leakage was 9.9% (*n* = 7) and 4.2% of patients (*n* = 3) exhibited necrosis of the gastric conduit. Seven patients had to undergo reoperation: three due to conduit necrosis, two due to chyle leak with retroperitoneal clipping of the chyle duct, one due to hemothorax and one for repositioning of an early enterothorax. The textbook outcome rate in the study cohort was 49.3% (35/71) (see Table 3).

**Table 3:** Postoperative outcomes

Postoperative outcomes	*n* = 71
ICU stay [days, median (IQR)]	1 (1–2)
Hospital stay [days, median (IQR)]	19 (15–26)
Clavien-Dindo-Classification [n (%)]	
< IIIa	50 (70)
IIIa	8 (11.3)
IIIb	4 (5.6)
IVa	7 (9.9)
IVb	1 (1.4)
V	1 (1.4)
Reoperation [n (%)]	7 (9.9)
Anastomotic leakage [n (%)]	7 (9.9)
Conduit ischemia [n (%)]	3 (4.2)
In-house mortality [n (%)]	1 (1.4)
90-day-mortality [n (%)]	1 (1.4)
30-day hospital readmission [n (%)]	5 (7.0)
Textbook outcome [n (%)]	35 (49.3)

A total of 26 patients (36.6%) were histopathologically evaluated as pT3, with 32 patients (45.1%) exhibiting nodal tumor manifestation. The median number of dissected lymph nodes was 20 (IQR: 17–27). 70 patients (98.6%) had negative resection margins. Complete tumor regression was observed in 15 patients (21.1%) (see Table 4).

**Table 4:** Oncological patient outcomes

Oncological outcomes	*n* = 71
Histopathological classification [n (%)]	
T0	18 (25.4)
Tcis	1 (1.4)
T1	16 (22.5)
T2	10 (14.1)
T3	26 (36.6)
T4	0
N0	39 (54.9)
N1	15 (21.1)
N2	8 (11.3)
N3	9 (12.7)
R0	70 (98.6)
R1	1 (1.4)
Complete regression [n (%)]	15 (21.1)
Number of dissected lymph nodes [n, median (IQR)]	20 (17–27)

### CUSUM analysis

**Fig. 1:**
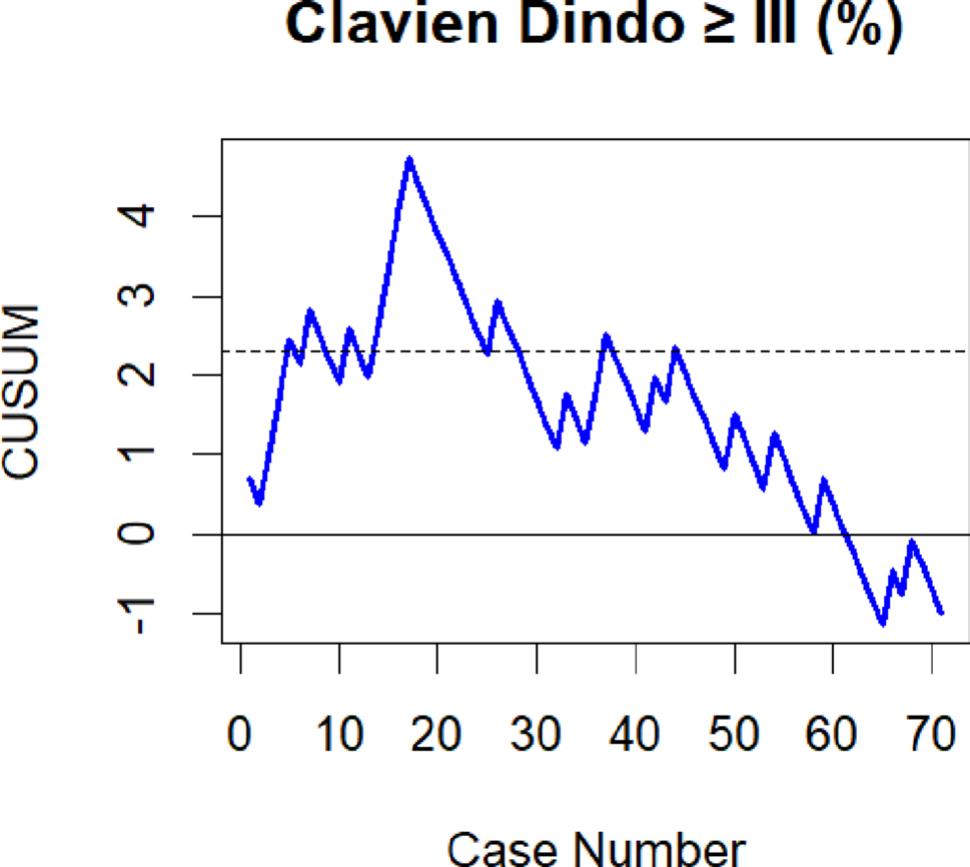
CUSUM analysis for Clavien Dindo complications ≥ III

For CD complications classified as IIIa or higher, an initial increase was observed in the CUSUM analysis until case 25, followed by a phase up to case 44, during which the curve fluctuated around the control limit (see Fig. 1). Subsequent to case 44, the process remained within the control limits, thereby indicating acceptable performance in relation to the complication benchmark. The major complication rates were 41.7% (10/24), 30% (6/20) and 18.5% (5/27) in these respective timeframes. However, the difference between complication rates during the early phase of the learning in comparison to the third phase did not reach statistical significance (*p* = 0.123; OR 3.07).

**Fig. 2:**
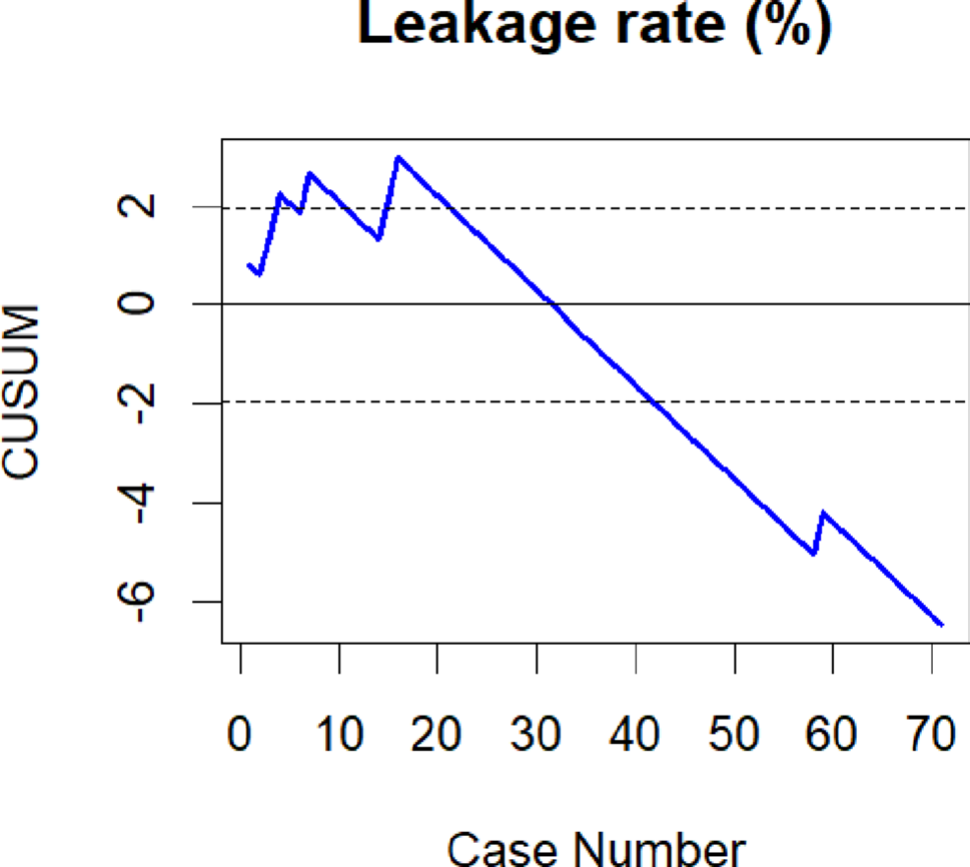
CUSUM analysis for anastomotic leakage

For anastomotic leakage, subsequent to an initial rise of the CUSUM plot, the LC remained within the control lines after case 20 (see Fig. 2). Eventually, from case 42 onwards it dropped below the lower control limit indicating a lower leakage rate in comparison to the chosen reference value of 18.6%. With 2%(1/51) the leakage rate after case 20 was significantly lower than during the initial phase of the LC (30% (6/20)) (*p* = 0.002; OR 20.3).

**Fig. 3:**
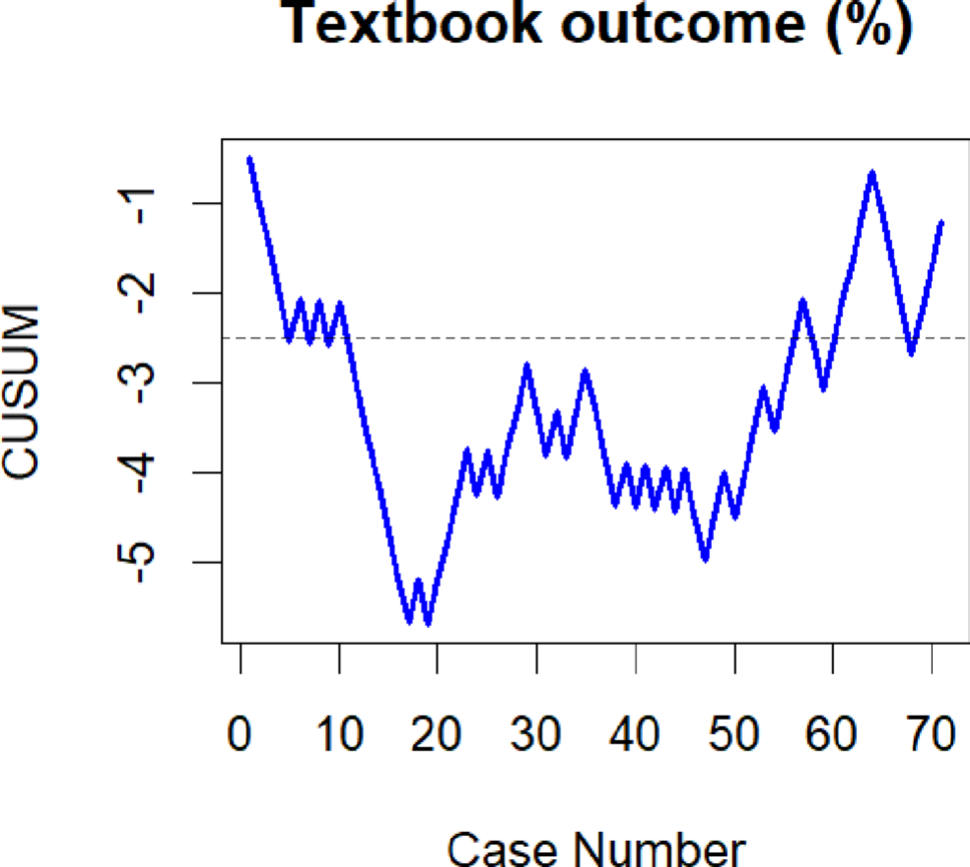
CUSUM analysis for textbook outcomes

For textbook outcome the LC initially declined below the lower control limit, signaling a lower rate of textbook outcomes in comparison to the chosen reference value. Subsequent to case 47, the CUSUM plot exhibited an upward trend, approaching the lower control limit and reaching it at case 54 (see Fig. 3).

**Fig. 4:**
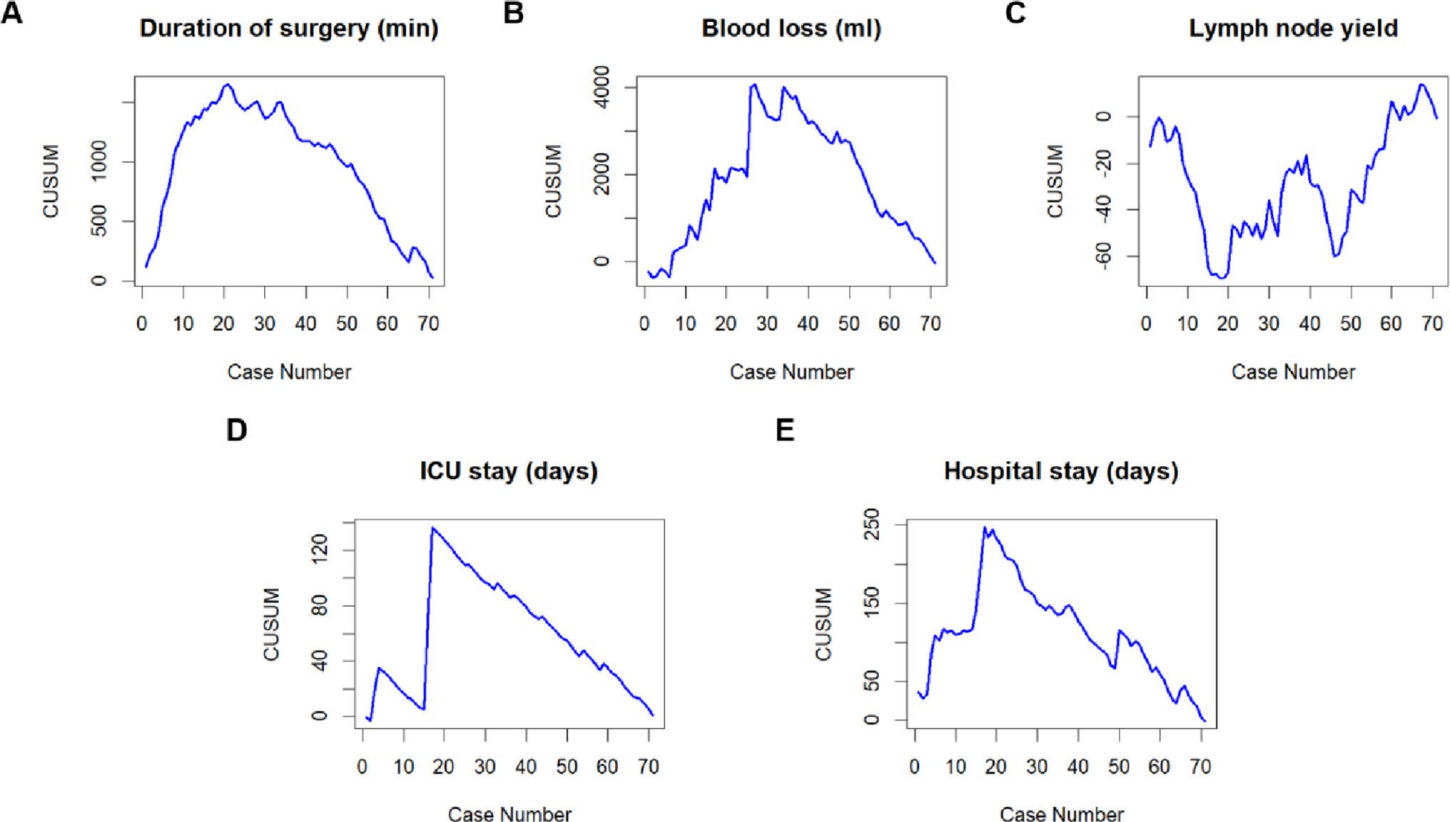
CUSUM analysis for intraoperative and postoperative parameters

The duration of surgery showed an initial increase, suggesting longer operation times in the beginning of the LC. Thereafter, a decline was observed, beginning after case 21 (see Fig. 4A). Similar results were found for intraoperative blood loss, ICU stay and hospital stay. Here, the curves descended after 34, 17 and 19 cases respectively (see Fig. 4B, D and E). The CUSUM for lymph node yield undulated during the initial 46 cases and started increasing afterwards (see Fig. 4C). The analysis of the parameters with statistical comparison of early and late stages of the respective LCs is shown in Table 5.

**Table 5:** Comparison of parameters during early and late phases of the learning curve as determined by the CUSUM analysis

	Inflection point (case no.)	Early phase	Late phase	*p*-value
CD ≥ III (%)*	44	41.7	18.6	0.123
Anastomotic leakage (%)	20	30	2.0	0.002
Textbook outcome (%)	NA	NA	NA	NA
Duration of surgery (min, mean)	21	383	271	< 0.0001
Blood loss (ml, mean)	34	560	332	0.005
Lymph node yield (no., mean)	46	20.4	24.1	0.047
ICU stay (days, mean)	17	12.2	1.7	0.001
Hospital stay (days, mean)	19	36.8	19.3	< 0.0001

Early phase was defined as cases up until the inflection point or crossing of the respective control limit and late phase as the following cases

*For CD the phase from case 25 to 44 was exempt due to the CUSUM undulating around the control limit

## Discussion

In the present study we performed an analysis for the LC of a new surgical team for their initial 71 cases of hRAMIE with intrathoracic circular stapler anastomosis at our institution. Given that the LC for this procedure is associated with relevant morbidity, we conducted a CUSUM analysis to investigate the effects of the introduction of this new team on the most relevant surgical outcome parameters [11]. In addition, we utilized the definition of textbook outcome established by Busweiler et al. to evaluate at what point during the LC these indicators for benchmark surgical performance can be reached. The incidence of complications CD ≥ IIIa, anastomotic leakage and textbook outcome in our cohort were compared to previously published historical cohorts. Furthermore, the duration of surgery, intraoperative blood loss, lymph node yield, ICU stay and length of hospital stay were analyzed in previously described fashion using an intrinsic mean for the CUSUM analysis [13].

As expected, the initial phase of the LC was associated with an increase in postoperative complication rates. However, the rate of CD complications classified as IIIa or higher quickly dropped from a rate of 41.7% during the first 24 cases of hRAMIE to 18.6% after completion of the LC from case 44 onwards. With an overall rate of postoperative complications CD ≥ IIIa of 29.6% during the study period, it was shown that benchmark complication rates can be reached within a brief time frame and are comparable to published reference values [22]. Concerning anastomotic leakage, the early phase of the LC was associated with an increase in complications. According to the CUSUM analysis a total of 20 cases of hRAMIE were necessary to overcome the LC for anastomotic leakages. For these initial 20 cases a significantly higher anastomotic leakage rate of 30% (6/20) was observed compared to the subsequent rate of 2% (1/51) after completion of the LC (*p* = 0.002; OR 20.3). With an overall leakage rate of 9.9%, the results from our study are comparable to the reported outcomes from experienced RAMIE centers after surmounting of the LC [21]. Fuchs et al. reported a similar LC of 38 cases for series of RAMIE with a robotic thoracic phase [15]. Concomitant to the described reduction in postoperative complication rates the duration of both ICU and hospital stay also significantly declined during the study period, with an LC of 17 and 19 cases, respectively. Furthermore, it was shown that textbook outcome can be reached using a hybrid approach in 49.3% of the cases during the LC. However, in order to achieve the benchmark rate for textbook outcome of 51% reported by the UGIRA study group a longer LC seems to be necessary. For instance, van Workum et al. reported a LC of 119 cases to achieve a textbook outcome rate of 54% in minimally invasive esophagectomy [11]. As the level of proficiency in hRAMIE increased, a concomitant reduction in the duration of surgery and the amount of blood loss was observed following cases 21 and 34, respectively. For lymph node yield a LC of 46 cases was needed to gain proficiency, with an average of 24 resected lymph nodes after completion of the LC, compared to 20 resected lymph nodes before (*p* = 0.047). Overall the reported LCs for the various outcome parameters in our study align with the reported range of 9 to 85 for RAMIE [12].

Our study demonstrated that hRAMIE can be taught safely using a step-up approach and an open thoracic anastomosis. In-house as well as 90-day mortality was 1.4% in our study population and comparable to the rate of 2.6% reported by Low et al. [22]. Nonetheless, previous experience in robotic surgery, especially for the supervising surgeons, is important in ensuring adequate guidance of the trainee during the LC. Especially, the application of double-console guidance seems favourable in this setting. Additionally, accurate preoperative screening of patient surgical history remains an important factor to ensure feasibility of minimally invasive abdominal resection. With conversion to laparotomy occurring in only one case this objective was achieved in our study.

In comparison to full minimally invasive esophagectomy, hybrid minimally invasive esophagectomy has been reported to be associated with a higher frequency of pulmonary complications, a higher leakage rate and lower lymph node count [23]. However, for comparison of these two techniques randomized controlled evidence remains scarce. On this topic, the results of the ongoing MICKey trial are awaited to answer the question if total RAMIE (tRAMIE) is superior to hRAMIE concerning overall postoperative complications [24]. Depending on the outcomes of this trial, hRAMIE could either serve as an alternative to tRAMIE or be introduced as a stepping stone during the establishment of a totally minimally invasive technique.

The presented study has several limitations. First, the data is from a single center including only one single surgical team performing the included procedures and was analyzed retrospectively reducing external validity. Second, for parameters like textbook outcome the LC could not be overcome during the study period. Third, outside comparison is limited by focus on operating time and lymph node yield as well as utilization of arbitrary statistical methods in other studies. Fourth, most studies focus on tRAMIE or hRAMIE with minimally invasive thoracic phase which hinders comparison of results to these studies. Furthermore, it is important to note the wide variety of different textbook outcome definitions in the literature [25]. In this publication, the Busweiler definition was utilized, as employed by the UGIRA, due to its high reference value and its capacity to facilitate comparison with the textbook outcome rate of the UGIRA’s benchmark. Nevertheless, the authors are aware of the existence of more recent consensus-based textbook outcome definitions [26].

Moving forward, we should target outcome variables like major postoperative complications and textbook outcome with external reference values through CUSUM analysis, incorporated into multicenter studies of training pathways in robotic programs.

## Conclusion

The findings of our study demonstrate that the implementation of a new surgical team for hRAMIE with a robotic abdominal phase can attain benchmark postoperative complication rates within a reasonable caseload. Textbook outcomes can be achieved at a high rate even during the LC.

## Supplementary Information

Below is the link to the electronic supplementary material.

Supplementary Material 1

## Data Availability

No datasets were generated or analysed during the current study.
