# Supplementary material for: Textbook outcome can be reached in the learning curve for hybrid robot-assisted esophagectomy–experience from a German high-volume center
Source: J Robot Surg. 2026 Apr 21;20(1):439. doi: 10.1007/s11701-026-03405-6 (PMC13095897; doi:10.1007/s11701-026-03405-6)
Supplement: Supplementary file 1 — Supplementary Material 1 [file 11701_2026_3405_MOESM1_ESM.docx]

Steps in full robotic Ivor-Lewis esophagectomy

1. Trocar placement easy
2. Cholecystectomy (optional) easy
3. Opening of the lesser sac easy
4. Gastrolysis (up to splenic hilum) easy
5. Gastrolysis (spleem to left crus) middle
6. Gastrolysis of pylorus middle
7. Lymphadenectomy Truncus challenging
8. Lymphadenectomy lesser curvature middle
9. Lymphadenectomy Hiatus challenging
10. Gastric tube formation middle
11. Preparation of V. azygos middle
12. Lymphadenectomy Aorta challenging
13. Lymphadenectomy Trachea challenging
14. Lymphadenectomy Pericard challenging
15. Cut-down esophagus middle
16. Pursue string suture challenging
17. Insertion of stapler middle
18. Pull-up of conduit challenging
19. Anastomosis stapling challenging
20. Resection of the specimen middle
